# Up-regulation of MARVEL domain-containing protein 1 (MARVELD1) accelerated the malignant phenotype of glioma cancer cells via mediating JAK/STAT signaling pathway

**DOI:** 10.1590/1414-431X2020e10236

**Published:** 2021-05-17

**Authors:** Lingyang Xia, Peng Jin, Wei Tian, Shuang Liang, Liye Tan, Binxin Li

**Affiliations:** 1 Department of Neurosurgery, The Second Affiliated Hospital of Mudanjiang Medical College, Mudanjiang, China; 2 Department of Operating Room, Hongqi Hospital Affiliated to Mudanjiang Medical College, Mudanjiang, China; 3 Department of X-ray, Hongqi Hospital Affiliated to Mudanjiang Medical College, Mudanjiang, China; 4 Department of Hepatobiliary Surgery, The Second Affiliated Hospital of Mudanjiang Medical College, Mudanjiang, China

**Keywords:** MARVELD1, Glioma, Prognosis, Functional analysis, JAK/STAT signaling pathway

## Abstract

This work aimed to research the function of MARVEL domain-containing protein 1 (MARVELD1) in glioma as well as its functioning mode. Bioinformatics analysis was utilized to assess the MARVELD1 expression in glioma tissues and its relationship with grade and prognosis, based on The Cancer Genome Atlas (TCGA), Genotype-Tissue Expression (GTEx), and Chinese Glioma Genome Atlas (CGGA) databases. Cell Counting Kit-8 (CCK-8), colony formation, and Transwell assays were carried out to determine the impact of MARVELD1 on malignant biological behavior of glioma, such as proliferation, invasion, and migration. qRT-PCR was carried out to test the mRNA level of MARVELD1. Western blot assay was performed to measure the protein expression of MARVELD1 and JAK/STAT pathway-related proteins. MARVELD1 was expressed at high levels in glioma tissues and cell lines. Kaplan-Meier survival analysis revealed that the higher MARVELD1 expression, the shorter the survival time of patients with glioma. Also, the MARVELD1 expression in WHO IV was significantly enhanced compared to that in WHO II and WHO III. Furthermore, the functional analysis of MARVELD1 *in vitro* revealed that knockdown of MARVELD1 in U251 cells restrained cell proliferation, migration, and invasion, while up-regulation of MARVELD1 in U87 cells presented opposite outcomes. Finally, we found that JAK/STAT signaling pathway mediated the function of MARVELD1 in glioma. MARVELD1 contributed to promoting the malignant progression of glioma, which is the key driver of activation of JAK/STAT signaling pathway in gliomas.

## Introduction

Glioma is the most common malignant tumor of the human central nervous system, and its extremely negative outcomes make it a major challenge to clinical treatment (1,2). Glioma has a high recurrence rate, and its invasive growth is considered to be the main cause of poor prognosis (3–5). With the continuous improvement of diagnosis and treatment technology, immunotherapy, gene therapy, and other new therapies have emerged (6–8). Although they provide a new direction for the treatment of glioma, the survival period of glioma patients has not been significantly prolonged (9). It has been found that many factors, including hypoxia microenvironment, oncogene overexpression, and over-activation of key signaling pathways, are involved in the invasive growth of malignant glioma (10–12). Hence, searching for effective molecular-targeted gene loci of glioma may open up new areas to conquer this disease.

MARVEL domain-containing protein 1 (MARVELD1) is a member of MARVEL family with vesicular transport and membrane connectivity (13). Proteins with MARVEL domain exert their function in many biological processes, including cell cycle process, chemotaxis, tight junction, memory grid mediated endocytosis, and others (14). It has been proven that MARVELD1 is lowly expressed in many cancers, such as cervical cancer, breast cancer, and prostate cancer (14,15). Concurrently, Alves et al. (16) found that MARVELD1 was expressed at high levels in colon cancer based on large-scale gene expression profile analysis. Of note, MARVELD1 expression in low-grade glioma is lower than that of high-grade glioma (according to the differentiation degree and proliferation potential of tumor cells of the World Health Organization classification), suggesting that MARVELD1 may play a role in glioma, but its function in glioma is still unknown.

Based on bioinformatics analysis, we revealed that MARVELD1 expression was strongly related to the JAK/STAT pathway. Herein, we assessed the MARVELD1 function in the malignant biological behavior of glioma cells as well as whether JAK/STAT pathway mediates this function, hoping to afford an effective biomarker for the clinical treatment of glioma.

## Material and Methods

### Bioinformatics analysis

The data of 168 glioma patients and 110 healthy controls, downloaded from The Cancer Genome Atlas (TCGA) and Genotype-Tissue Expression (GTEx) databases, were used to analyze the differential expression of MARVELD1. The data from the Chinese Glioma Genome Atlas (CGGA) website (http://www.cgga.org.cn) (mRNAseq_325, mRNAseq_693, mRNA_array_301) were utilized to analyze the clinical information of the glioma patients. Kaplan Meier plots were built to measure whether the survival rate was related to the MARVELD1 expression. The pathways related to MARVELD1 expression were enriched by gene set enrichment analysis (GSEA), using the high *vs* low MARVELD1 expression.

### Culture and treatment of cell lines

Human glioma cell lines U87 and U251 were obtained from the Cell Bank of the Chinese Academy of Sciences (China). Normal human astrocytes (NHA), purchased from Sciencell Research Laboratories (USA), were used as control. Dulbecco's Modified Eagle's medium (DMEM) was utilized to culture all cells at 37°C with 5% CO_2_, which contains 10% fetal bovine serum (FBS), 100 U/mL penicillin, and 0.1 mg/mL streptomycin.

For up-regulation of MARVELD1, the overexpression vector pcDNA3.1- MARVELD1 was purchased from Shanghai GenePharma Co., Ltd. (China). Also, siRNA sequences targeting for MARVELD1 were designed for down-regulation of MARVELD1. si-MARVELD1#1 (5′-CCACCTTAAGATTCCCAGCATC-3′), si-MARVELD1#2 (5′-GCTGTGTTCTGTTTGAAGATTC-3′), and si-con (5′-AATTCTCCGAACGTGTCACGT-3′) were synthesized by Shanghai GenePharma Co., Ltd. All transfections in this study were performed by Lipofectamine 2000 (Invitrogen, USA) following the manufacturer's protocol. After 48 h, the transfected cells were harvested for subsequent experiments.

### Quantitative RT-PCR

Total RNA in cells was isolated with TRIzol reagent (Invitrogen), and then reverse transcribed RNA into complementary DNA using Reverse Transcription Kit (TaKaRa, China), based on the standard. SYBR Premix Ex Taq (TaKaRa) was used to detect the mRNA level of MARVELD1 with the following primers: MARVELD1-F: 5′-CGCTGGCTCATGGTCAACGTG-3′, MARVELD1-R: 5′-CCTTGAGGTTGCAGTAGCTGTG-3′, following the manufacturer's protocol. GAPDH (F: 5′-GTCTCCTCTGACTTCAACAGCG-3′, R: 5′- ACCACCCTGTTGCTGTAGCCAA-3′) was used for normalization and the 2^−ΔΔCT^ method was used to compute the data.

### Western blotting analysis

Cells were lysed to extract the protein with RIPA lysate (contains protease inhibitor), and the protein concentration was measured using Bicinchoninic Acid kit (Sigma-Aldrich, USA). Sodium dodecyl sulfonate-polyacrylamide gel (10%) electrophoresis (SDS-PAGE) was used to separate the lysate, and the gel was transferred to the polyvinylidene fluoride (PVDF) membrane, which was then blocked in 5% skimmed milk powder for 1 h. After that, primary antibodies were used to immune-stain the membranes at 4°C. After overnight incubation at 4°C, the membranes were cultivated with the secondary antibody at room temperature. The images were developed with electrochemical luminescence and the bands were quantified using ImageJ software (NIH, USA).

### Cell Counting Kit-8 (CCK-8) assay

Cells were seeded in 96-well plates with 2000 cells/well, at 24, 48, and 72 h after cultivation, and the viability of transfected cells was detected using CCK-8 kit following the manufacturer's instructions (Dojindo Molecular Technologies, USA). The absorbance was analyzed at 450 nm using a microplate reader.

### Colony formation assay

Cell populations were released by trypsin, and then inoculated and cultured at 37°C with 5% CO_2_ in a culture dish (containing 5 mL pre-heated medium) for 7-14 days. When the visible clone appeared, the cells were fixed in 4% paraformaldehyde and stained with crystal violet.

### Transwell assays

Cellular migration and invasiveness were assessed in Transwell chambers. The transfected cells, suspended in serum-free medium, were added in the upper chamber (BD, USA) pre-coated with Matrigel (invasion assay) or without Matrigel (migration assay), and the medium with 10% FBS was filled to the matched lower chamber as a chemoattractant. After incubation for 24 h, the cells on the upper surface of the filters were removed, while the cells that migrated or invaded through the chamber were fixed and stained with crystal violet. Then, cells were counted in five fields of view.

### Statistical analysis

Statistical analysis was performed using SPSS 20.0 software (IBM, USA). All assays were performed 3 times, and the data are reported as means±SD. The statistical significance of data was determined using Student's *t*-test (two groups) or one-way ANOVA with Dunnett's post-test of (three groups and above). The overall survival was assessed using Kaplan-Meier survival analysis, based on the median expression level of MARVELD1. P<0.05 was considered that the difference was statistically significant.

## Results

**Figure 1:**
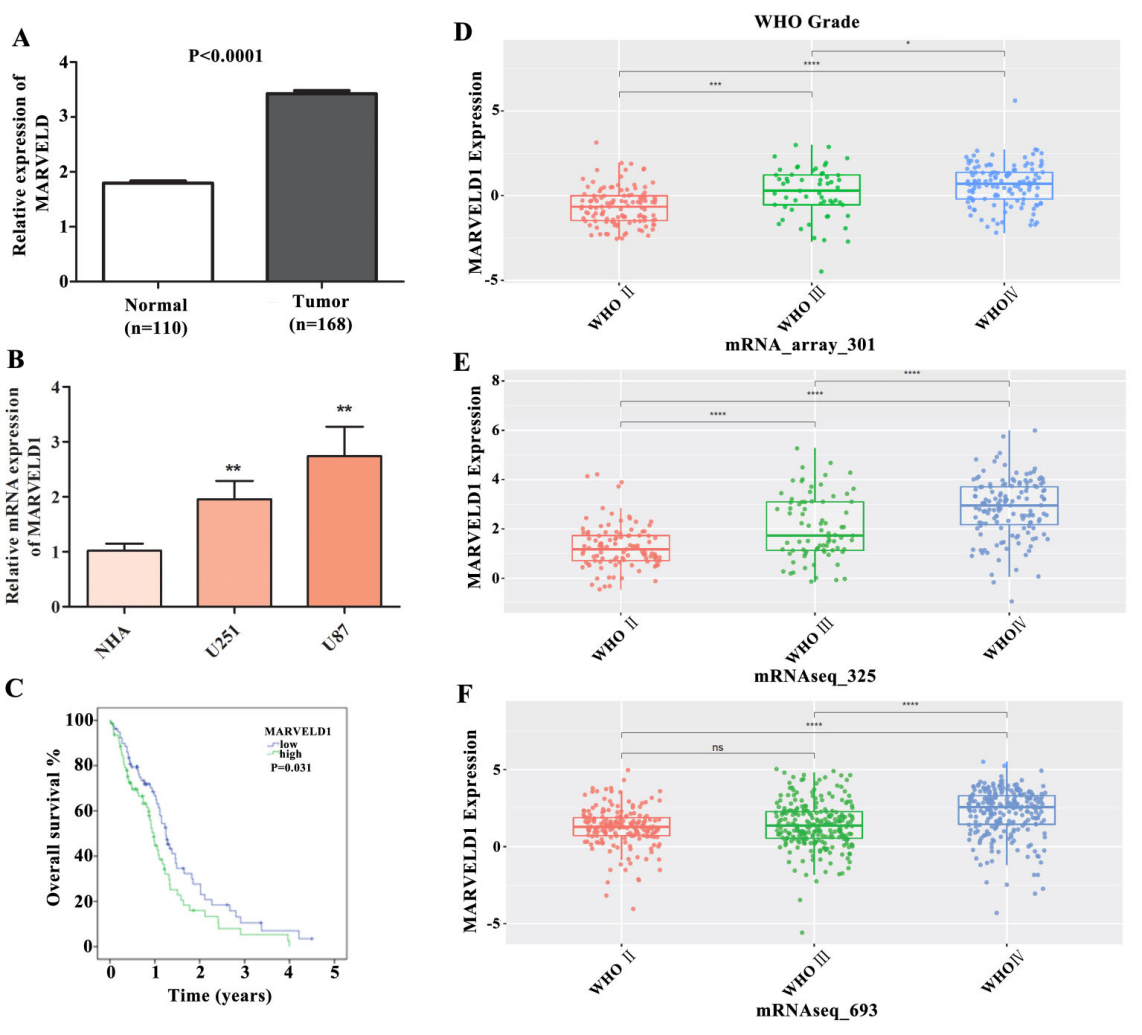
MARVELD1 was expressed at high levels in glioma and correlated with prognosis and WHO grade. **A**, The differential expression of MARVELD1 in glioma patients (n=168) and healthy controls (n=110) were analyzed based on the TCGA and GTEx databases. **B**, MARVELD1 expression in glioma cell lines (U87 and U251) and control (NHA) was measured using qRT-PCR. **C**, The relationship between the MARVELD1 level (high or low) and the survival time of glioma patients was assessed using Kaplan Meier analysis. **D**-**F**, MARVELD1 expression in WHO stage II-IV glioblastoma was analyzed based on CGGA databases (mRNA_array_301, mRNAseq_325, mRNAseq_693). Data are reported as means±SD. **P<0.01 (Student's *t*-test or one-way ANOVA with Dunnett's post-test).

**Figure 2:**
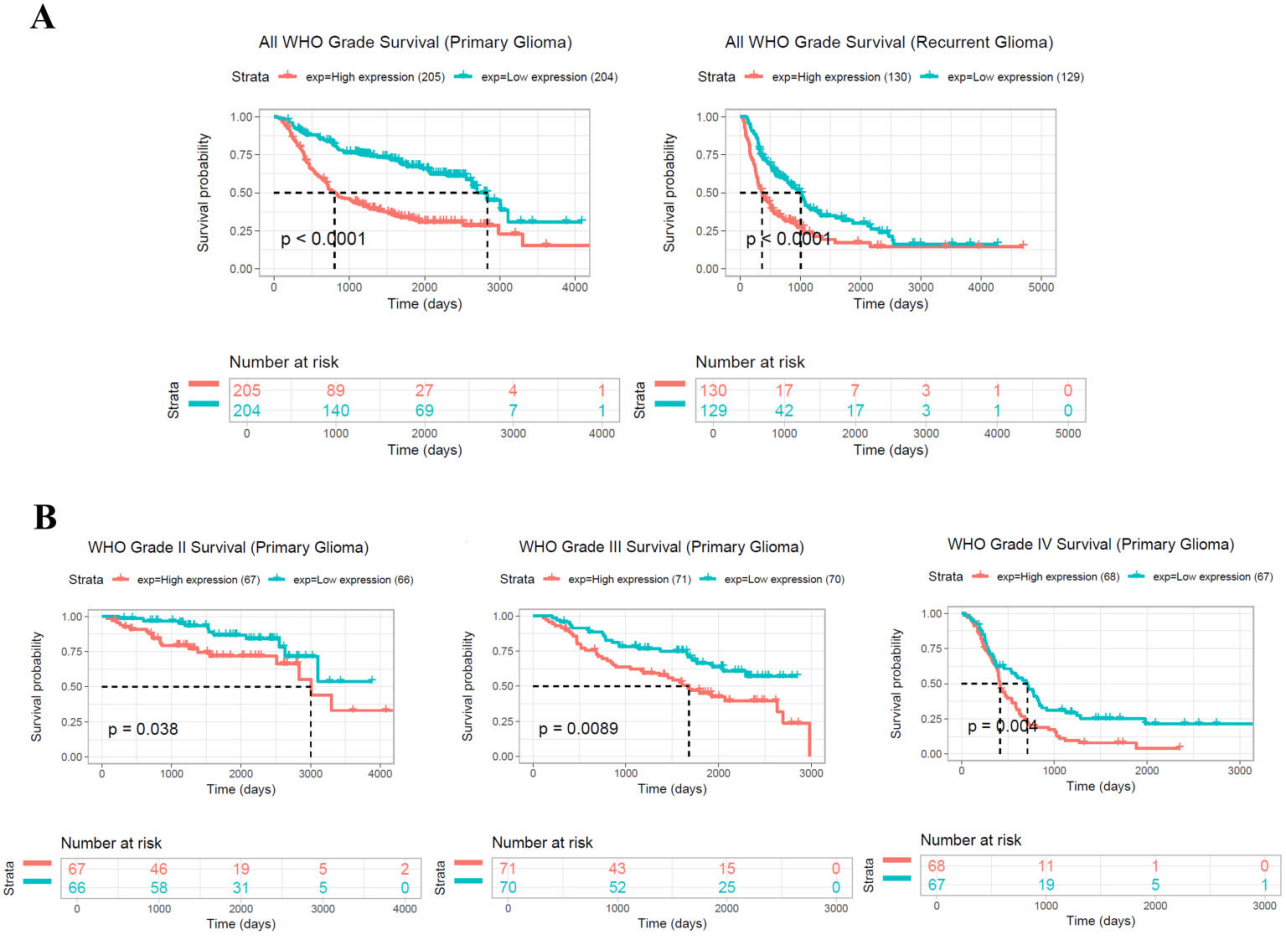
The higher the MARVELD1 expression, the lower the survival rate in all WHO II, WHO III, and WHO IV grades. **A**, Survival curves of primary glioma and recurrent glioma based on all WHO grades. **B**, Survival curves of primary glioma based on WHO II, WHO III, and WHO IV grades. Data are reported as means±SD. P<0.01 (Student's *t*-test or one-way ANOVA with Dunnett's post-test).

MARVELD1 was expressed at high levels in glioma and high MARVELD1 expression was related to prognosis and WHO grade (range I-IV). Based on the data in the TCGA and GTEx databases, we found that MARVELD1 expression was markedly enhanced in glioma tissues compared to that in the normal control (Figure 1A). Then, to identify the expression of MARVELD1 in glioma cell lines U87 and U251, qRT-PCR was performed. As expected, MARVELD1 expression was increased in both U87 and U251 cells compared to the normal cell NHA (Figure 1B). Kaplan Meier plots revealed that high MARVELD1 expression in glioma was related to poor outcomes (Figure 1C). Furthermore, the connection between MARVELD1 expression and clinicopathological parameters was analyzed based on the CGGA database. As presented in Figure 1D-F, there was a significant correlation between high MARVELD1 expression and WHO grade. A higher grade indicated a higher MARVELD1 expression, and the MARVELD1 expression in WHO IV was significantly enhanced compared to that in WHO II and WHO III. Moreover, the survival curves based on WHO grades were also analyzed. As a result, the higher the MARVELD1 expression, the lower the survival rate in all WHO II, WHO III ,and WHO IV grades (Figure 2A and B).

**Figure 3:**
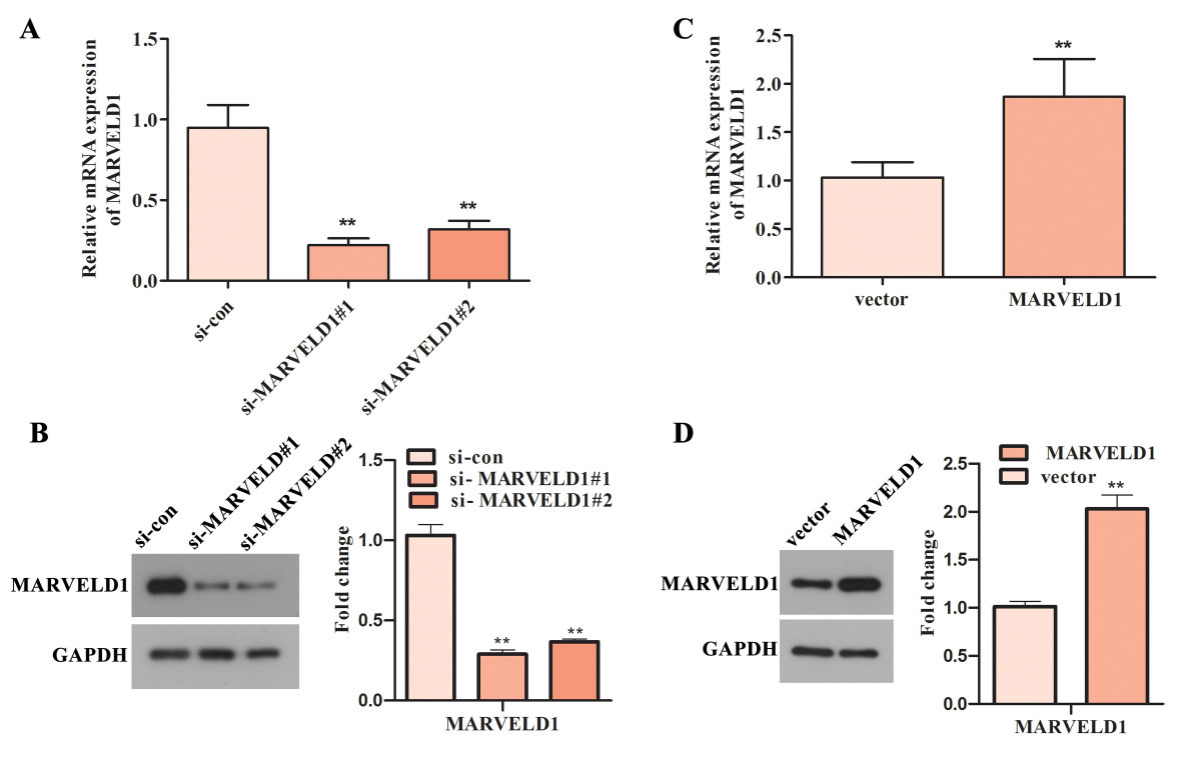
The transfection efficiency of si-MARVELD1 in U251 cells and pcDNA3.1-MARVELD1 in U87 cells. **A** and **B**, The mRNA and protein levels of MARVELD1 in si-MARVELD1-treated U251 cells were detected using qRT-PCR and western blot assays. **P<0.01 *vs* si-con group. **C** and **D**, qRT-PCR and western blot assays were also performed to assess the mRNA and protein levels of MARVELD1 in pcDNA3.1-MARVELD1-treated U87 cells. **P<0.01 *vs* vector group. Data are reported as means±SD (Student's *t*-test or one-way ANOVA with Dunnett's post-test).

Knockdown of MARVELD1 was done by si-MARVELD1 in U251 cells and overexpression of MARVELD1 was done by pcDNA3.1-MARVELD1 in U87 cells. To better assess the role of MARVELD1 in glioma, U87 cells, which presented a relative lower expression, were selected for overexpression assays, and U251 cells with a relative higher expression were chosen for knockdown assays. U87 cells were transfected with pcDNA3.1-MARVELD1 to upregulate MARVELD1, and U251 cells were transfected with si-MARVELD1#1 and si-MARVELD1#2 to downregulate MARVELD1. As presented in Figure 3A and B, si-MARVELD1#1 and si-MARVELD1#2 caused a significant decrease in the mRNA and protein levels of MARVELD1 in U251 cells. In addition, si-MARVELD1#1 with relative higher interference efficiency was used in the subsequent experiment. On the contrary, increased mRNA and protein levels of MARVELD1 were found after U87 cells were treated with pcDNA3.1-MARVELD1 (Figure 3C and D). All these data indicated that the transfection was successful in both U87 and U251 cells.

**Figure 4:**
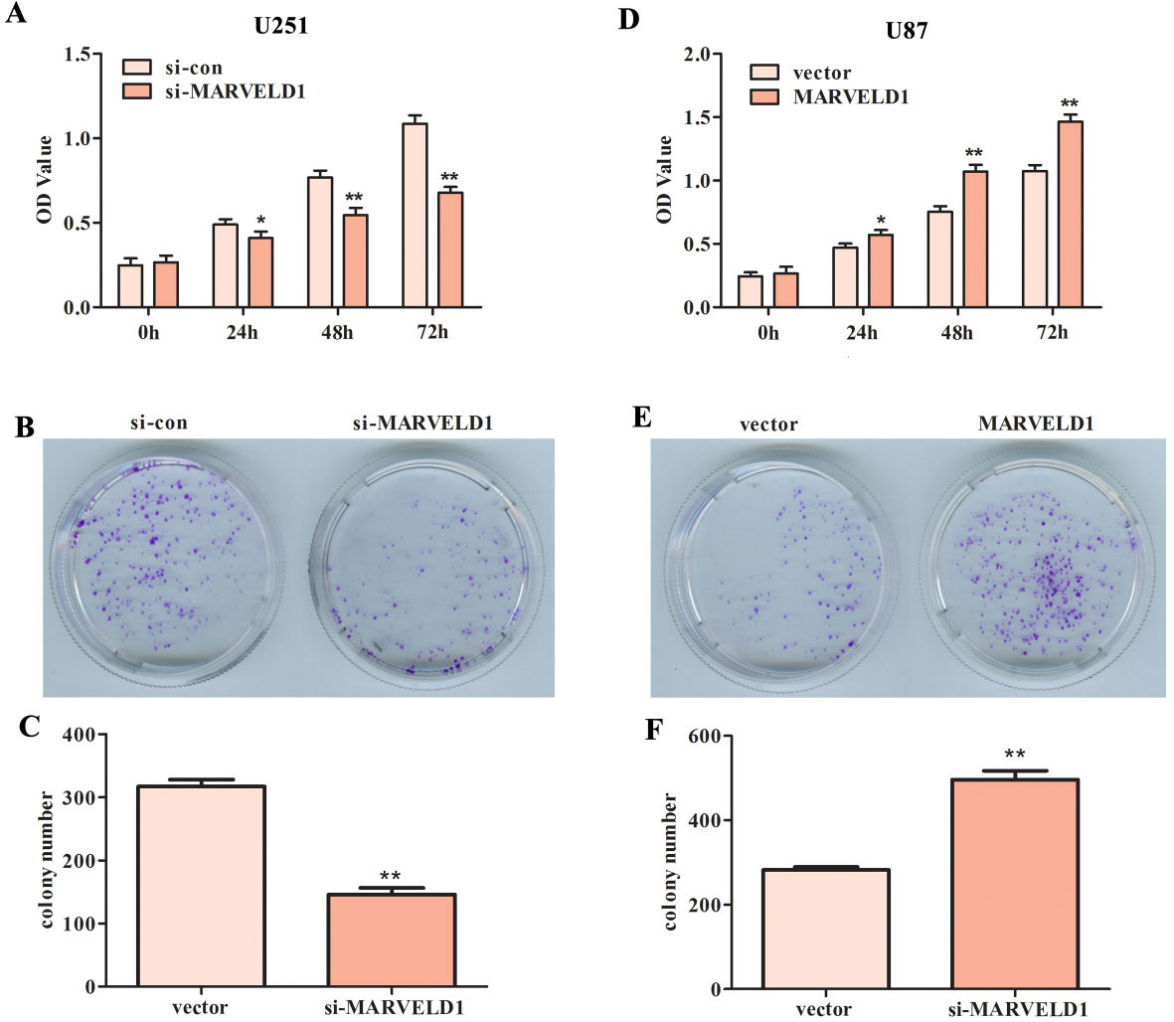
Down-regulation of MARVELD1 in U251 cells suppressed proliferation and up-regulation of MARVELD1 in U87 cells led to a promotion in proliferation. CCK-8 assay and colony formation assay were conducted to measure the cell viability and the proliferative capacity after knockdown of MARVELD1 in U251 cells (**A**-**C**), and after overexpression of MARVELD1 in U87 cells (**D**-**F**). Data are reported as means±SD. **P<0.01 *vs* vector group (Student's *t*-test).

CCK-8 analyses revealed that cell viability clearly decreased in U251 MARVELD1-lacking cells (Figure 4A), while upregulation of MARVELD1 increased the viability of U87 cells (Figure 4B). The colony formation assay presented similar results. The U251 cells had decreased proliferation after transfection with si-MARVELD1 compared to control (Figure 4C and D), while colony number was significantly increased with up-regulation of MARVELD1 in U87 cells (Figure 4E and F). These results revealed that MARVELD1 accelerated glioma cell proliferation *in vitro*.

**Figure 5:**
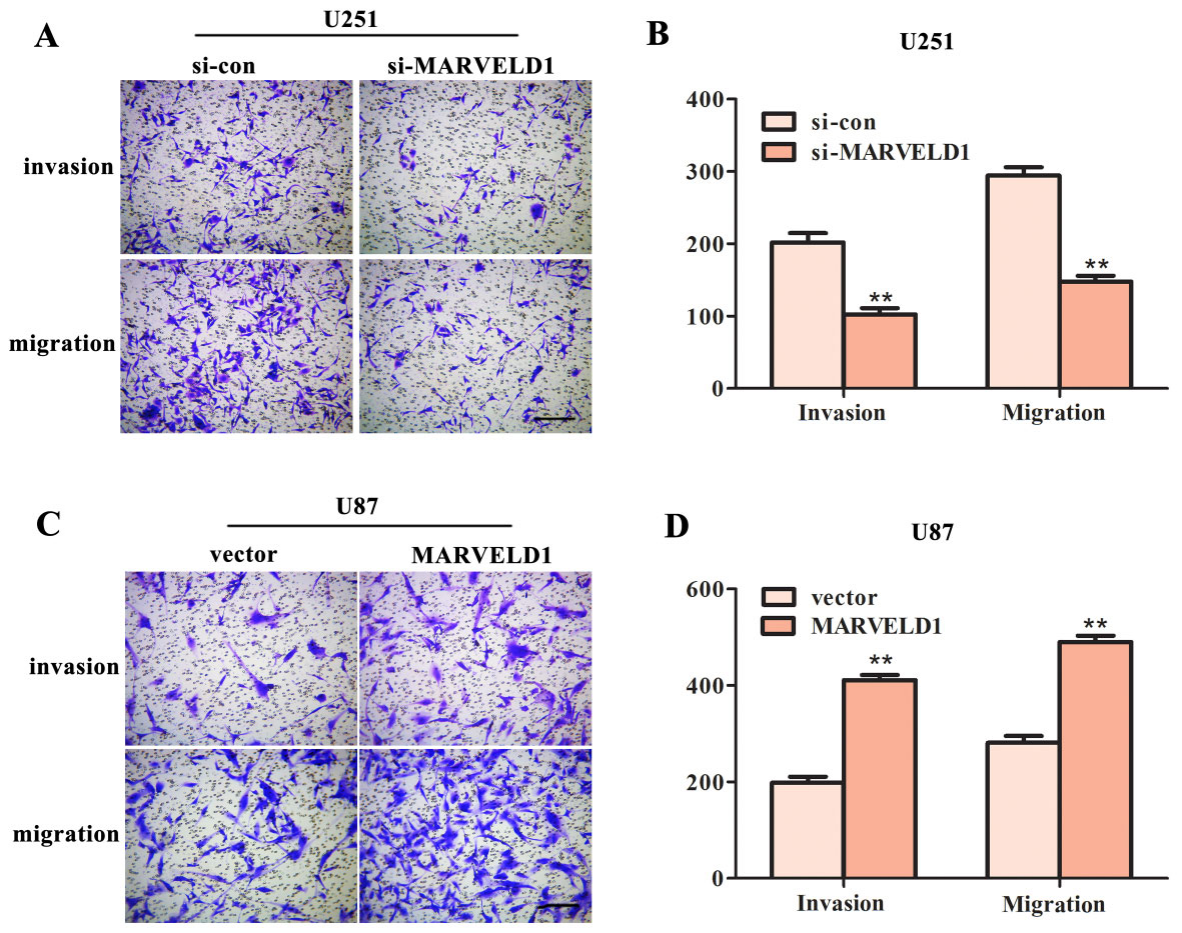
**A** and **B**, Transwell assay was carried out to measure the invasive and migratory abilities of U251 cells transfected with si-MARVELD1. **P<0.01 *vs* si-con group. **C** and **D**, Invasion and migration of U87 cells transfected with pcDNA3.1-MARVELD1 were detected using Transwell assay. Magnification bars: 200 µm. Data are reported as means±SD. **P<0.01 *vs* vector group (Student's *t*-test).

MARVELD1 promoted the invasion and migration of glioma cells. Considering that MARVELD1 enhanced the proliferation of glioma cells, we next performed the Transwell assay to evaluate the potential function of MARVELD1 in the invasive and migratory abilities of glioma cells. As can be seen in Figure 5A and B, suppression of MARVELD1 was able to clearly decrease the number of migrated and invaded U251 cells, whereas U87 with up-regulated MARVELD1 presented a significant increase in invasion and migration (Figure 5C and D). These data demonstrated that MARVELD1 may be essential for the invasion and migration of glioma cells.

**Figure 6:**
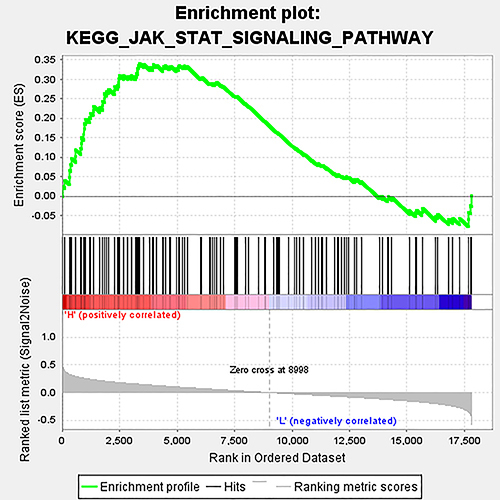
GSEA analysis was carried out to identify the pathways closely related to MARVELD1 expression.

**Figure 7:**
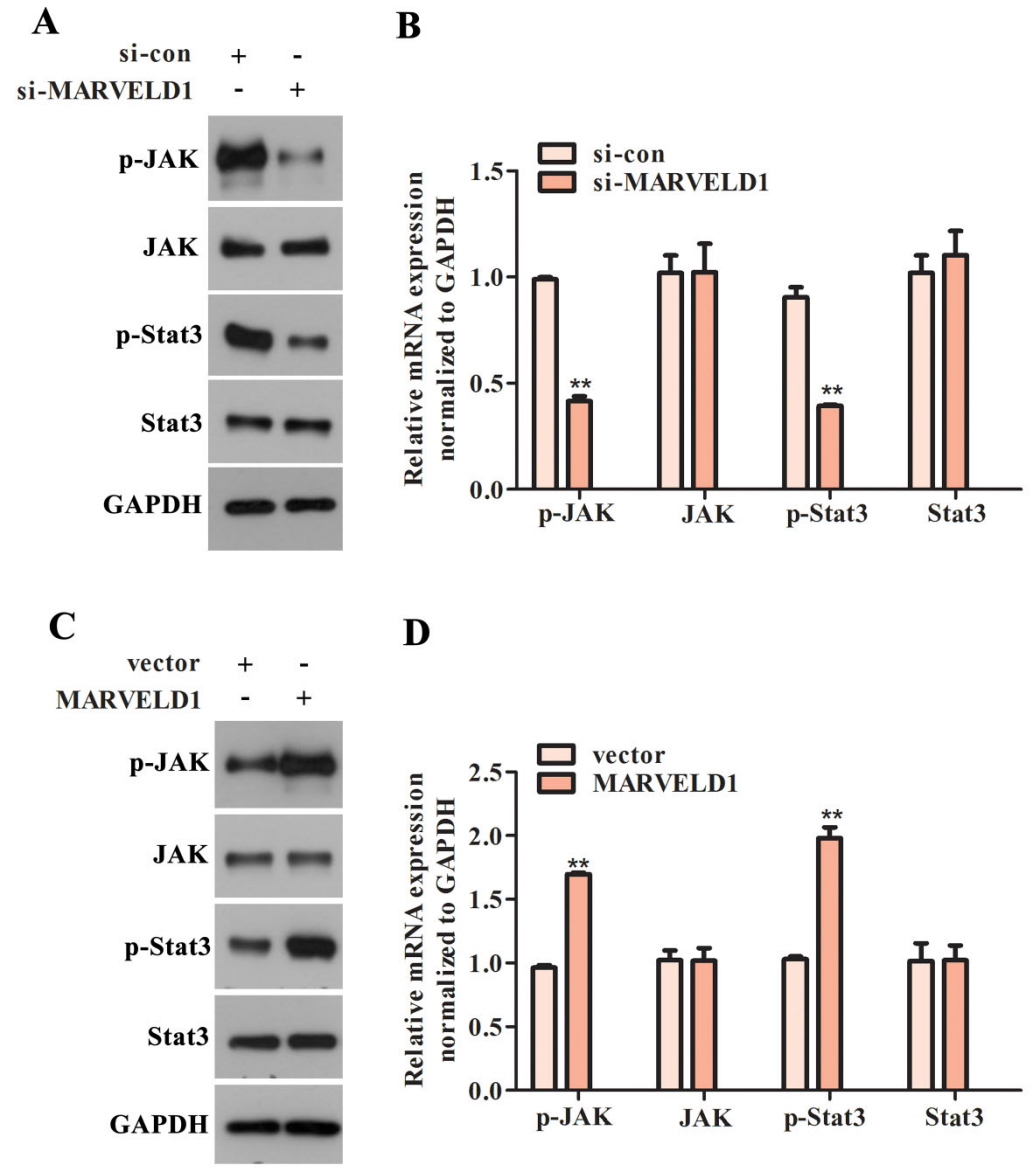
Western blot assay was performed to measure the expression levels of JAK/STAT pathway-related factors in (**A** and **B**) U251 cells transfected with si-MARVELD1 (**P<0.01 *vs* si-con group) and (**C** and **D**) U87 cells transfected with pcDNA3.1-MARVELD1. Data are reported as means±SD. **P<0.01 *vs* vector group (Student's *t*-test).

JAK/STAT pathway mediates the modulation of MARVELD1 on the biological behavior of glioma cells. Next, GSEA analysis was performed to discern the possible MARVELD1-associated pathways in glioma. The results indicated that the expression of MARVELD1 was strongly related to the JAK/STAT pathway (Figure 6). According to the aforesaid result, we forecasted that MARVELD1 may facilitate the malignant progression of glioma through JAK/STAT signaling pathway. Then, we performed western blot assay to detect the JAK/STAT-pathway-related proteins. The phosphorylation levels of JAK and Stat3 were significantly decreased after U251 cells were transfected with si-MARVELD1, while the total JAK and Stat3 expression was almost unchanged (Figure 7A and B). Contrarily, using pcDNA3.1-MARVELD1 in U87 cells caused a significant increase in the phosphorylation levels of JAK and Stat3 (Figure 7C and D). All these data showed that the promoting impact of MARVELD1 on the malignant progression of glioma may be achieved via JAK/STAT signaling pathway.

## Discussion

Malignant transformation of tumors is often accompanied by abnormal gene expression. In this present work, we found for the first time that MARVELD1 was up-regulated in glioma, and patients with high expression of MARVELD1 presented a shorter survival time. Also, we found that MARVELD1 was correlated to grade, and the level of MARVELD1 in patients with high-grade glioma was higher than that in patients with low-grade glioma. Additionally, up-regulation of MARVELD1 contributed to accelerating the malignant biological behavior of glioma cells, such as proliferation, invasion, and migration, and this function may be achieved through JAK/STAT pathway.

MARVELD1 is located on human chromosome 10q24, which functions in the proliferation and migration of tumor cells and participates in the malignant progression of various cancers (14,17,18). Previously, MARVELD1 has been studied for its anti-cancer effect, for instance, MARVELD1 inhibits the proliferation of malignant cells in liver cancer (19) as well as suppresses EMT in non-small cell lung cancer (17). MARVELD1 is highly expressed in colon cancer (16) and male germ cell tumor, hinting that the role of MARVELD1 in cancer may be a double-edged sword. In this paper, MARVELD1 was up-regulated in glioma and associated with poor outcomes and grade. Gliomas are classified as WHO I-IV following the classification of central nervous system tumors by the World Health Organization in 2016 (20). Here, patients with WHO IV glioma presented higher MARVELD1 expression compared with WHO II and WHO III gliomas, and higher MARVELD1 expression resulted in shorter survival time. Also, based on the bio-functional assays, MARVELD1 promoted the malignant phenotype of glioma cells. All these findings indicated that MARVELD1 was involved in the malignant progression of glioma.

How the function of MARVELD1 in glioma is realized needs further study. GASE analysis showed that JAK/STAT signaling pathway was closely related to the expression of MARVELD1. Previously, extensive studies have found that JAK/STAT signaling pathway contributed to many basic biological processes such as cell proliferation, apoptosis, angiogenesis, and immune response (21–23). In recent years, it has been found that activation of JAK/STAT signaling pathway plays a significant role in tumor development, especially STAT3 (24). STAT3 functions in promoting cell cycle progression and preventing cell apoptosis (25,26). Notably, the activation of JAK/STAT pathway caused a poor outcomes in glioma patients (27). Che et al. (28) found that repression of miR-30 can restrain JAK/STAT3 pathway and decrease the carcinogenicity of glioma stem cells. Our findings revealed that up-regulation of MARVELD1 enhanced the phosphorylation of JAK and STAT3, leading to JAK/STAT signaling pathway activation. Since JAK/STAT signaling pathway is a key molecule involved in tumor cell proliferation, invasion, and migration (29,30), we believed that MARVELD1 functions in glioma at least partially through JAK/STAT signaling pathway.

In summary, our results indicated that MARVELD1 exerted a tumor-promoting impact on the malignant phenotype of glioma cells via activating JAK/STAT signaling pathway *in vitro.* It could be a potential effective target for glioma therapy, but these findings are limited to glioma cell lines *in vitro*, and the function of MARVELD1 in glioma *in vivo* needs further research.
